# An intelligent framework to measure the effects of COVID-19 on the mental health of medical staff

**DOI:** 10.1371/journal.pone.0286155

**Published:** 2023-06-08

**Authors:** Muhammad Irfan, Ahmad Shaf, Tariq Ali, Maryam Zafar, Saifur Rahman, Meiaad Ali I. Hendi, Shatha Abduh M. Baeshen, Maryam Mohammed Mastoor Maghfouri, Hailah Saeed Mohammed Alahmari, Ftimah Ahmed Ibrahim Shahhar, Nujud Ahmed Ibrahim Shahhar, Amnah Sultan Halawi, Fatima Hussen Mahnashi, Samar M. Alqhtani, Bahran Taghreed Ali M.

**Affiliations:** 1 Electrical Engineering Department, College of Engineering, Najran University, Najran, Saudi Arabia; 2 Department of Computer Science, COMSATS University Islamabad, Sahiwal Campus, Sahiwal, Pakistan; 3 Armed Forces Hospital Jazan, Jazan, Saudi Arabia; 4 Armed Forces Hospital Southern Region, Khamis Mushait, Saudi Arabia; 5 Ministry of Health, Riyadh, Saudi Arabia; 6 Al-Twal General Hospital, Al-Twal, Jazan, Saudi Arabia; 7 Department of Information Systems, College of Computer Science and Information Systems, Najran University, Najran, Saudi Arabia; Jeonbuk National University, KOREA, REPUBLIC OF

## Abstract

The mental and physical well-being of healthcare workers is being affected by global COVID-19. The pandemic has impacted the mental health of medical staff in numerous ways. However, most studies have examined sleep disorders, depression, anxiety, and post-traumatic problems in healthcare workers during and after the outbreak. The study’s objective is to evaluate COVID-19’s psychological effects on healthcare professionals of Saudi Arabia. Healthcare professionals from tertiary teaching hospitals were invited to participate in the survey. Almost 610 people participated in the survey, of whom 74.3% were female, and 25.7% were male. The survey included the ratio of Saudi and non-Saudi participants. The study has utilized multiple machine learning algorithms and techniques such as Decision Tree (DT), Random Forest (RF), K Nearest Neighbor (KNN), Gradient Boosting (GB), Extreme Gradient Boosting (XGBoost), and Light Gradient Boosting Machine (LightGBM). The machine learning models offer 99% accuracy for the credentials added to the dataset. The dataset covers several aspects of medical workers, such as profession, working area, years of experience, nationalities, and sleeping patterns. The study concluded that most of the participants who belonged to the medical department faced varying degrees of anxiety and depression. The results reveal considerable rates of anxiety and depression in Saudi frontline workers.

## Introduction

The World Health Organization discovered at the end of 2019 that pneumonia was driven on by an unforeseen factor in Wuhan city, in the Chinese province of Hubei. At the beginning of 2020, World Health Organization declared the disease a pandemic due to the worldwide growth of the coronavirus (COVID-19) [1]. Additionally, the World Health Organization designated COVID-19 a global health emergency unprecedented in the modern age. It affected the physical and psychological health of the general public and caused severe levels of anxiety, stress, and depression [2]. The situation poorly affected Italy’s population, which recorded more than 31000 casualties and over 223000 infected with COVID-19 diseases [3].

Research on past epidemics shows severe stress and anxiety levels in front-line health workers. In contrast, the diseases of COVID-19 affected the psychological and physical health of healthcare workers badly [4]. The study has revealed that working participants are more inclined towards COVID-19 with a score of 2.0 and standard deviation of 0.5. The ratio of infection in medical staff serving from front line is relatively high in comparison to those who are working in other departments [5]. In comparison with other healthcare workers, nurses shows a less percentage of stress or psychological disorders. Among those who did not have psychological symptoms, a higher proportion of nurses were observed (40.2% out of n = 194), compared to doctors (29.5% out of n = 142) and non-medical healthcare workers (30.3% out of n = 146) [6].

A study conducted a metavariable analysis which states that physical medical condition and prior health condition are kept as independent predictor for COVID-19 irrespective of the participating country and non healthcare personnel [7]. Similary, a study has revealed tat during outbreak of COVID-19, there is an important correlation between psychological disordered and prevalence of physical symptoms in healthcare workers. Furthermore, it also states the symptoms as bi-directional and suggest timely interventions for healthcare with physical symptoms. 48 (5.3%) tested positive for moderate to very-severe depression, 79 (8.7%) for moderate to extremely-severe anxiety, 20 (2.2%) for moderate to extremely-severe stress, and 34 (3.8%) for moderate to severe levels of psychological distress among the total of 906 participants. 33% of the participant s were found with more than four symptoms and 33.4% reported a frequent headache symptoms [8].

Women were tend to have a negative relation with score “Avoidance of disclosure and discrimination related to COVID-19” domain (Coefficient. = -0.27, CI: -0.43 to -0.12), but a positive relation with the “Negative attitude towards working conditions” domain (Coefficient. = 0.19, CI: 0.09 to 0.3). Furthermore, working in administrative offices (Coefficient. = 0.20; 95% CI = 0.05 to 0.36) and infectious departments (Coefficient. = 0.36; 95% CI = 0.09 to 0.63) showed a positive relation with the “Increased work pressure due to COVID-19” domain [9]. According to research in China and Italy, the global pandemic has no exceptions regarding the mental health of medical staff [10].

According to previous research, infectious diseases left severe continuous, and long-lasting psychopathological consequences on the medical staff. SARS (severe acute respiratory syndrome), which came out in 2003, affected front-line health workers. The health professional reported a shortage of encouragement at work and showed cognitive signs of severe stress [11]. Similarly, in Middle East Respiratory Syndrome (MERS), which erupted in 2015, the doctors were reported to develop PTSD (Post-Traumatic Stress Disorder), which caused an increase in absences from work [12].

By April 2020, COVID-19 had infected almost 12,000 medical professionals, and 228 doctors and 26 nurses had passed away [13]. Due to limited contact with official psychological support, a lack of personal medical information on the outbreak, and inadequate training on personal protective equipment and infection control protocols, non-frontline healthcare workers are also at risk of experiencing elevated stress [14].

Numerous researchers from all over the world studied the COVID-19-related mental health problem. The study, carried out in China, revealed that more than half of the respondents had medium to extreme anxiety, and 50% of those surveyed said they had medium to extreme psychological symptoms [15]. In [16], 19.6% of respondents claimed to have experienced anxiety ranging from moderate to severe at the time of the COVID-19 epidemic. Higher depression levels were associated with being a female student, living with a COVID-19-susceptible family member, being unmarried or separated, and being a student.

In [17], 2081 inhabitants and citizens of Saudi Arabia were examined to see how the pandemic affected their mental health. 7.3% of the respondents indicated that they experienced anxiety, according to the findings. The researchers also concluded that non-Saudis, single parents, senior citizens, and college students were the most likely to suffer from depression during the epidemic. A higher level of worry was associated with Saudi individuals who are married, unemployed, and wealthy.

In this study, we have analyzed the survey that was conducted to collect information from the health line workers of Saudi Arabia. The study aims to predict the levels of anxiety and prediction by using the data provided in the survey. The purpose of this study is to determine the prevalence of mental symptoms and highlight the factors that are causing anxiety and depression in healthcare workers. The contribution of this paper is as follows:

- The critical impression of the study is the comprehensive review of the health workers’ data and the impact of the COVID-19 outbreak on their mental health.
- The proposed study utilized a number of machine learning algorithms, and the results that are being carried out from these models are satisfying.
- The machine learning models utilize the sample data and predict the risk factors that are more likely to contribute to causing depression and anxiety among healthcare workers.
- The results may assist the government agencies and Healthline Professionals in assessing the contributing factors for the depression and anxiety of the medical staff.

The rest of the paper is organized as follows; section 2 shows the related work, section 3 represents the materials and method of the proposed work, section 4 displays the findings of proposed algorithm on the dataset, and finally concludes the paper in the conclusion section.

## Methodology

**Fig 1:**
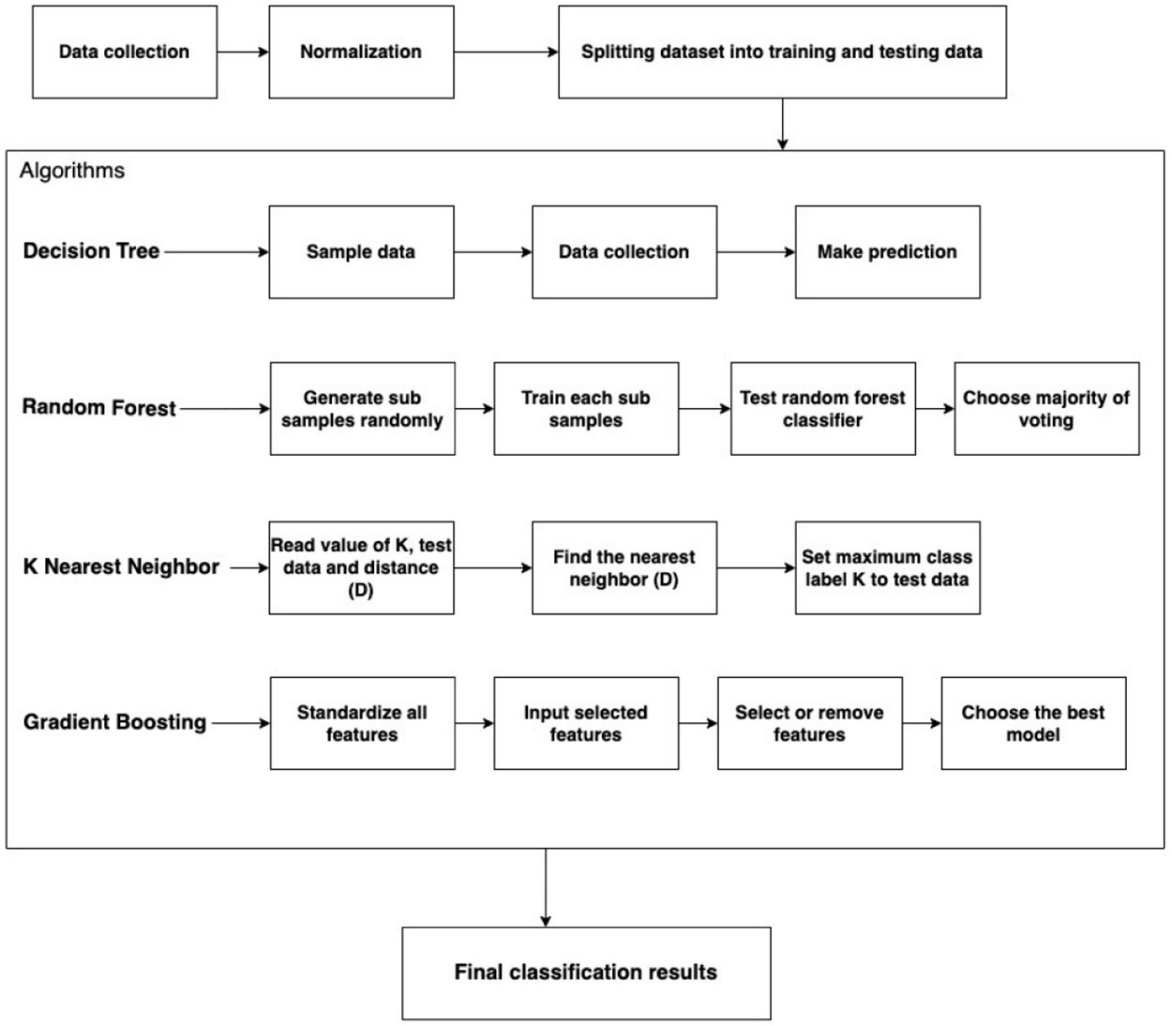
Intelligent framework of the proposed methodology.

In order to assess the concern of health workers, past behavior, knowledge, and attitude were observed to decide the psychological impact of COVID-19 on their mental health. That is why similar questions were asked during the data collection in Saudi Arabia. Several general questions were included in the survey covering different aspects such as modes of COVID-19, symptoms, transformation, and several prevention metrics. The dataset uses a scale to measure the anxiety and depression level of the respondent. The study uses classification techniques and different relevant algorithms such as DT (Decision Tree), RF (Random Forest), KNN (K nearest neighbor), GB (Gradient Boosting), LightGBM (Light gradient boosting machine), and XGBoost (extreme gradient booster) to predict the impact of COVID-19 on the psychological state of Health Care Workers (HCW) as shown in Fig 1. The results are carried out in Precision, Recall, F1-score, and Accuracy.

### Dataset details

The utilized dataset in the study is obtained from the given link (https://www.ncbi.nlm.nih.gov/pmc/articles/PMC8418795/bin/peerj-09-12119-s001.sav). The dataset contained numerous gaps, and the data quality was not satisfactory to apply a machine-learning algorithm. The quality of data directly impacts the quality of the model. As the dataset contains different types of information, it is likely for vital information to be amidst the noise in the vast feature space. As the dataset contains numerical and categorical data, it is transformed into the most appropriate structure to collect maximum accuracy. Several data scrubbing techniques and feature engineering methods are being applied on datasets to encode non-numeric data, deal with missing values, handle outliers and transform data in a format acceptable by the algorithm. Data cleaning aims to develop a dataset that answers research questions and can give appropriate results. The categorical features in the dataset are separated from the other columns. The results showed that there are 58 categorical features in the dataset. The findings then proceed with different quartiles, means, and maximum values. The feature info with the detail is obtained and exhibits 57 non-null columns in the dataset. The data is then proceeded to preprocessing, and feature engineering is applied to shape the data in a format that makes a model building can be possible.

The study used a questionnaire dataset that analyzed the behavior of different respondents who participated in the survey. The survey includes participants from different clinical departments and units of hospitals, including intensive care units, emergency departments, and general wards. The survey used a convenient technique in the selection of participants. The survey also explained its purpose prior to the participants via electronic mail so that they could show a keen interest in the activity and tend to share the correct information. The respondents were also given the reliability of asking questions before contributing to the survey. The utilized dataset is the result collected by the response of different people who work in different departments of healthcare centers and have served during the period of COVID-19. The data was analyzed statistically, and the Likert scale base question assisted in summarizing the analytics. Physicians and non-physicians are separated in the dataset with the help of Fisher’s exact test. In order to assess the impact of different columns in the prediction, the study undergoes classification analyses considering the type of health care facility, ID, Age, Age record, gender, nationality, profession, work area, year of experience, sleeping disorder before COVID-19, mental disorder, sleeping pills, trouble to stay awake, enthusiasm, sleep quality, bed partner, loud snoring, log pause, leg twitching, disorientation, anxiety, level of anxiety, depression, and level of depression.

**Fig 2:**
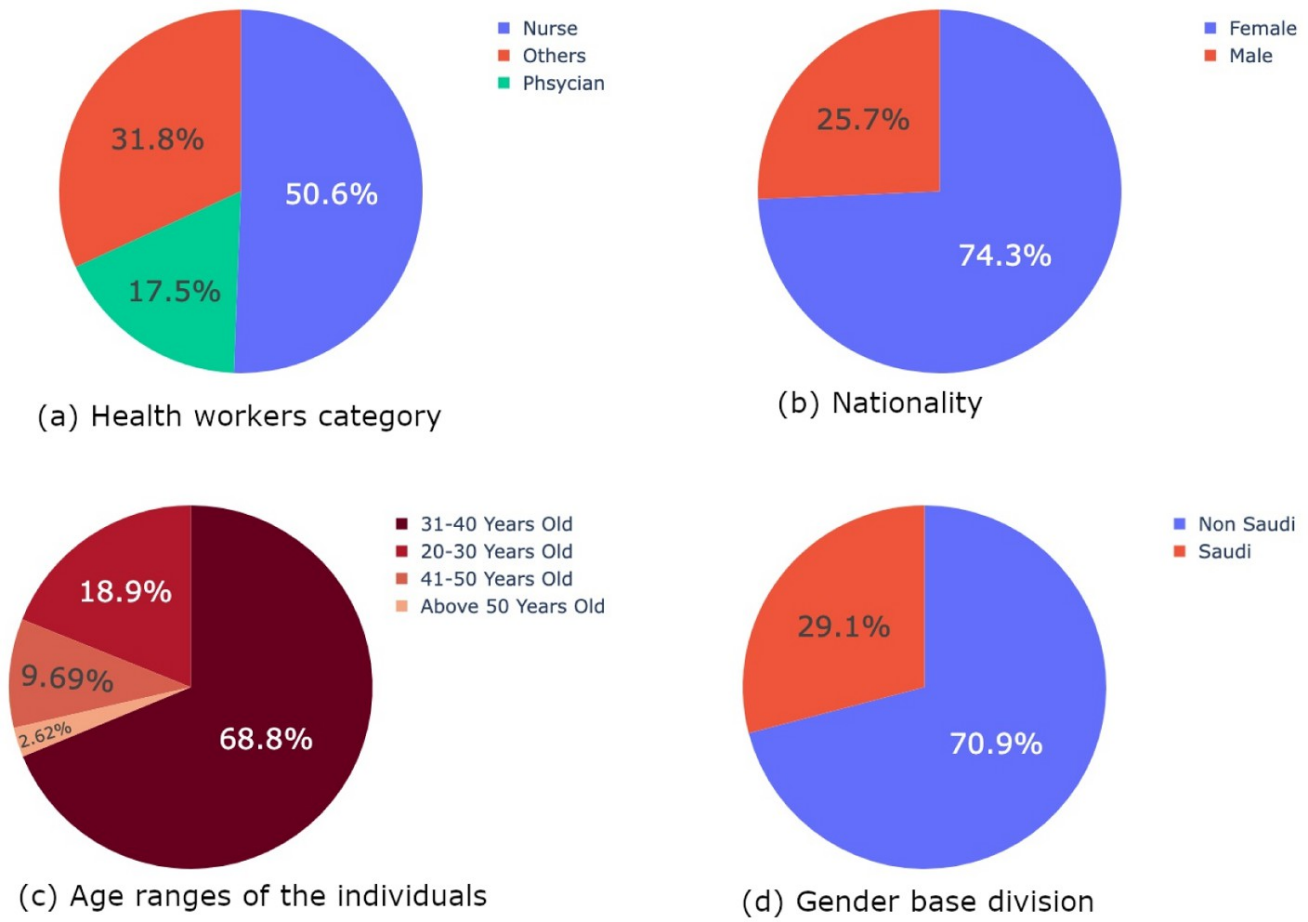
Feature engineering applied on different variable. (a) Health workers category. (b) Nationality. (c) Age ranges of the individuals. (d) Gender base division.

The demographics also analyze the age of the participants. The dataset has explained three professions of health care workers: physicians, nurses, and others as shown in Fig 2(a). The demographics of the dataset show that the ratio of nurses and midwives involved in the process is the highest at 50.6%. The dataset has also depicted the working area of the participants. The columns of working units take into account four working areas: ER, ward, ICU, and others. The demographics show that the ratio of acute care units (ER and ICU) is 44.8%. In contrast, other working areas, such as general hospital floors, auxiliary services, outpatient clinics, and academics, show 19.4%, 4.5%, 28%, and 3.3%, respectively.

The dataset considers the ratio of both genders who responded to the survey. A pie graph is plotted to analyze the ratio of male and female respondents in the survey as shown in Fig 2(b). The ratio of female respondents in the survey is high in comparison to male respondents. The Blue area depicts the female participants, while the red area depicts the male participants. Around 74.3%, 483 of the females have responded to the survey, and the detail of age, profession, year of experience, and working area is added to the dataset. While 25.7%, 127 of the males, have participated in the survey, the age group, working experience, profession, and other credentials can be seen in the dataset.

A pie chart is plotted to analyze the response rate based on nationalities. The red part in the pie chart depicts the ratio of Saudi participants, while the blue area exhibits the ratio of non-Saudi participants as depicted in Fig 2(c). The ratio for non-Saudi participants is at 70.9%, which is 461. In contrast, the ratio for Saudi participants is comparatively low at 29.1%, which is 189.

Similarly, a pie chart is plotted to analyze the number of age groups included in the dataset as described in Fig 2(d). The response rate for 31 to 40 years is the highest. A total of 447, 68.8%, have participated in this age group. The second highest age group in the dataset is 20 to 30 years old. Of 123, 18.9% have been included in this age group. Similarly, the age group of 41 to 50 also gave satisfactory results. The response rate from this age group is 9.69% which is 63 in number. The respondents from the age scale above 50 are the lowest. Only 2.62%, 17 in number, have participated in the survey.

### Decision tree

Due to visualization simplicity, decision trees are considered well-known machine learning algorithms. A tree is constructed in the decision trees, which are divided into different nodes. These nodes help in figuring out the hierarchy of the model and the overall importance of features at first glimpse. A dataset is broken down into smaller pieces in the decision tree. These smaller pieces consist of statistics with a higher homogeneity. As the dataset contains different features, the constructed decision tree will be of different kinds with different depths for the training model. However, building an optimal decision tree with the shortest length is computationally expensive and can predict the class label of any unseen or new data [18].

The decision tree has three nodes: root node, leaf node or terminal node, and internal node. The class label is the leaf node in the decision tree classifier. The feature test conditions fall into the category of a leaf or terminal nodes. The hierarchy of non-terminal nodes plays a crucial role in order to build an optimal decision tree. Decision tree assists in feature selection in this study as it works perfectly to predict the significance of each attribute. A decision tree helps split the sample appropriately, which is helpful in order to reduce the entropy of the dataset sample after that subset. The entropy of the sample is given as follows:

$$\begin{array}{c} E(S)=-\sum_{}^{}ni=1\rho i\;\text{log}\;(\rho i) \end{array}$$ (1)

Where Pi denotes the entropy of class S., A simple decision tree is used in the mental health dataset to give the accuracy. It also helps analyze the model’s most efficient predictor [19].

### Random forest

Random forest is an ensemble technique that generates various classifiers and combines their output. Random forest assists in creating different classification and regression trees (CART). Original training data is used to train each CART sample, and splits are determined with the help of a random search across a subset of input variables. A node is split into a child node repeatedly and generates the CART, the binary decision tree. The splitting is initiated with the root node, consisting of the entire learning sample [20]. The random forest classifier performs by casting a vote against each input. Most of the selected inputs in the random forest will be considered as the outcome. Random forest helps in the ensemble of a large number of trees, and they can manage highly dimensional data [21]. The following features of the random forest have helped in the proposed study:

- The random forest has an efficient feature to estimate the missing values in the dataset.
- Random forests help balance the imbalanced data in the data set with its significant feature of weighted random forest (WRF).
- The random forest also highlights the importance of variables used in the classifier.

### K nearest neighbor

K nearest neighbor (KNN) is famous among several machine learning algorithms because of its simplicity. It focuses on the concept that if objects are near each other, they probably share similar interests. It helps in classifying the object with the majority of the votes. The object is then kept in the class that is the most relevant among KNN. K is the small integer in the method and is usually upbeat. The algorithm measures the distance from the new data point to all the known data points. This unique observation belongs to the nearest class in the neighboring set. KNN is the best choice when there is little or no hint about primary data. However, suppose the dimension of the feature vector is high. In that case, KNN becomes computationally expensive as it needs to keep knowledge of all the data points and their relevant distance for the new data observation [22].

### Gradient boosting

Many individual decision tree models are combined to build a gradient tree-boosting model. The error made by the previous trees can be minimized by fitting the trees, and decision trees are collected by adding one at a time. The differentiable loss functions are minimized in this model with the help of gradient or stochastic gradient descent. The gradient boosting algorithm is preferred over several other machine learning algorithms in the tabular dataset. For the gradient-boosting ensemble model, the total amount of decision trees involved in the process is one of the crucial hyperparameters. The ensemble model improves the algorithm’s performance with the efficient combination of the total number of trees and the depth of the trees. Gradient boosting helps augment multiple weak tree-based classifiers in machine learning and builds superior and highly efficient results from these classifiers.

On the other hand, it is also different from the random forest in functionality, as the random forest utilizes precious data to train the trees in sequential order. The gradient boosting model generated new trees at each stage of the model, which aims to reduce the error made by the previous tree. It ensures the improvement of the accuracy of the model. As a non-linear algorithm, it is helpful to outperform the datasets with a high relationship compared to linear algorithms. There are several other types of gradient boosting involving XGboosting, CAT boost, and LightGBM, but all these types differ in the mechanism and implementation of the boosted trees [23].

### Light gradient boosting

Light Gradient Boosting Machine (LightGBM) is a popular learner mainly applied in boosting frameworks. Unlike CatBoost, it sequentially trains the data. Gradient-based one-side sampling (GOSS) is used in LightGBM because it ensures the optimal balance between enhanced speed by minimizing the number of data points. It holds the overall preciseness of the learned trees in this process. The ability to work with one-side sampling based on a gradient and exclusive feature combining highlights the performance of LightGMB from CatBoost and XGBoost. GOSS utilizes a slight gradient to rescue a considerable proportion of data samples. The rest of the data instances predict the information gain in single decision trees. LightGBM has proved that large data instances hold a significant role in the computational process of information gain. GOSS achieves a more accurate prediction of the information gained with the smaller data size. Exclusive feature bundling helps in reducing the number of features. Nonzero values are rarely included in mutually exclusive features, such as the single hot encoding feature. According to LightGBM, it IS NP-hard to figure out the best combination of unique features. A greedy algorithm such as LightGBM can assist in achieving a better estimation ratio. It works without affecting the overall model’s accuracy and also helps reduce the features [24].

### Xtreme gradient boosting

XGBoost or extreme boosting is the further implementation of gradient boosting algorithms. Extreme gradient boosting is considered the outperforming model when utilized in supervised learning. It is the most widely used algorithm as it performs in both classification and regression techniques. The computational behavior of XGBoost is out of the core, and its execution speed is considerably high compared to other algorithms [25]. Both single and distributed systems can be used in XGBoost as it supports parallel processing. It is widely used for large datasets as it efficiently manages the memory exceeding RAM. The number of regularizations in XGBoost helps in reducing overfitting. It also assists in determining a specific size of the decision tree and supports tree pruning, which means a decision tree will not grow after a specific limitation. XGBoost is also helpful in estimating missing values in the dataset. Initially, XGBoost was utilized in the machine learning models and GBM to enhance the training time. Several multiclass classification difficulties can be solved by utilizing a parallel tree-boosting framework. Instead of typical training and testing, it prefers cross-validation, considered a well-known technique for finding optimal accuracy. The data is shuffled randomly in the cross-validation method. The dataset is divided into k groups, each considered for testing data. The rest of the data is used to learn the model to find better accuracy. The remaining data also assist in measuring and delivering the k-fold average of accuracy. Leave one outcross validation is obtained by assigning the number of k to the number of observations [26].

## Results

The dataset was collected with the assistance of 71.8% of Health Care Workers (HCWs) who helped in gathering information. Among the respondents, 75% were female, and a majority of 62.4% belonged to the nursing profession. The machine learning algorithms’ performance was evaluated using precision, recall, and F1-score as indicators. Precision helped to determine the accuracy of the prediction made by the model by measuring how close the predicted value was to the actual value. On the other hand, recall indicated the usefulness of the model in predicting positive samples. A higher recall value meant the model could detect more positive samples.

Table 1 shows the prediction values for depression detection using different machine learning models. The proposed machine learning model included Decision Tree, Random Forest classifiers, K nearest neighbor, and LightGBM, which had the highest recall values. Accuracy was used to assess how similar the predicted value was to the actual value. F1-score was used as a measure of the harmonic mean of precision and recall. The binary classification system can be evaluated with the help of the F1-score. Higher F1-score means the model is performing generally better. In the proposed machine learning model, the Decision tree, Random Forest Classifier, K nearest Neighbor, and LightGBM have the highest F1-score. These algorithms outperform the research and offer the most satisfactory results. The precision rates for the decision tree are above 97%, which is reasonably satisfactory. Random forest classifier and K nearest Neighbor are also successful in providing the most favorable outcomes, such as 95% rates for precision, recall, and F1-score, respectively. It is sometimes challenging to predict results with gradient-boosting algorithms. Training takes longer in gradient boosting classifiers as they are prone to overfitting and intensive to the resources. However, in this research, Light Gradient Booting Classifier has offered a considerable outcome of 99%. LightGBM outperforms all the other algorithms and gives the best rates for accuracy.

**Table 1:** Level of depression detection.

Model	Precision	Recall	F1-Score
Decision Tree	0.97	0.97	0.97
Random Forest Classifier	0.95	0.95	0.95
K Nearest Neighbour	0.95	0.95	0.95
Gradient Boosting Classifier	1.00	1.00	1.00
Light Gradient Boosting Classifier	0.99	0.99	0.99
Extreme Gradient Boosting Classifier	1.00	1.00	1.00

In addition to depression detection, the study also explored predicting anxiety levels in health workers. The dataset collected participants’ anxiety and depression levels during and after the Covid-19 pandemic, where anxiety was defined as a feeling of fear and loss of control under a particular situation. The results showed that health workers had a high level of anxiety during the pandemic, while depression signs were still evident even after the pandemic period. Table 2 presents the prediction results for anxiety levels using six different algorithms. The Light Gradient Boosting Classifier had the highest accuracy among the six algorithms, with nearly 99% accuracy in predicting both depression and anxiety levels. Higher precision, recall, and F1-score values indicated higher prediction rates. The Random Forest Classifier and K Nearest Neighbor also offered similar results, with an accuracy rate of almost 98%. The Decision Tree and Extreme Gradient Boosting Classifier had a result of 1, while the F1-score ranged between 0 to 1, with values closer to 1 indicating better results for prediction. The overall accuracy rate of the model was above 99%, making it suitable for further use.

**Table 2:** Level of anxiety detection.

Model	Precision	Recall	F1-Score
Decision Tree	1.00	1.00	1.00
Random Forest Classifier	0.98	0.98	0.98
K Nearest Neighbour	0.98	0.98	0.98
Gradient Boosting Classifier	1.00	1.00	1.00
Light Gradient Boosting Classifier	0.99	0.99	0.99
Extreme Gradient Boosting Classifier	1.00	1.00	1.00

Performance measures help in analyzing the overall results of the machine-learning model. The amalgamation of results is represented in the confusion matrix of different algorithms, which visually presents findings. Four primary classification attributes are being utilized as performance measures in this study: Accuracy, Precision, Recall, and F1-Score are described in Eqs 2–5. If both the actual value and predicted value are positive, True Positive (TPV) result is classified. If both the actual and predicted values are negative, then the result is classified as True Negative (TNV). If the actual value is negative and the predicted value is positive, then the result is classified as False Positive (FPV). Similarly, if the predicted value is negative and the actual value is positive, the results are classified as False Negative (FNV). The model’s accuracy can be calculated when all the actual values are divided by the predicted values and the actual prediction.

Similarly, an equation calculates precision, dividing the true positive value by both actual positive and false positive values. The equation for calculating recall is similar to precision; it divides true positives with actual positive and false negative values. F1-score can be calculated with the help of the following equation. It multiplies precision and recall by two and divides it by adding both to get results.

$$\begin{array}{c} Accuracy=\frac{TPV+TNV}{TPV+TNV+FPV+FNV} \end{array}$$ (2)

$$\begin{array}{c} Precisin=\frac{TPV}{TPV+FPV} \end{array}$$ (3)

$$\begin{array}{c} Recall=\frac{TPV}{TPV+FPV} \end{array}$$ (4)

$$\begin{array}{c} F1-Measure=\frac{2*Precision*Recall}{Precision+Recall} \end{array}$$ (5)

**Fig 3:**
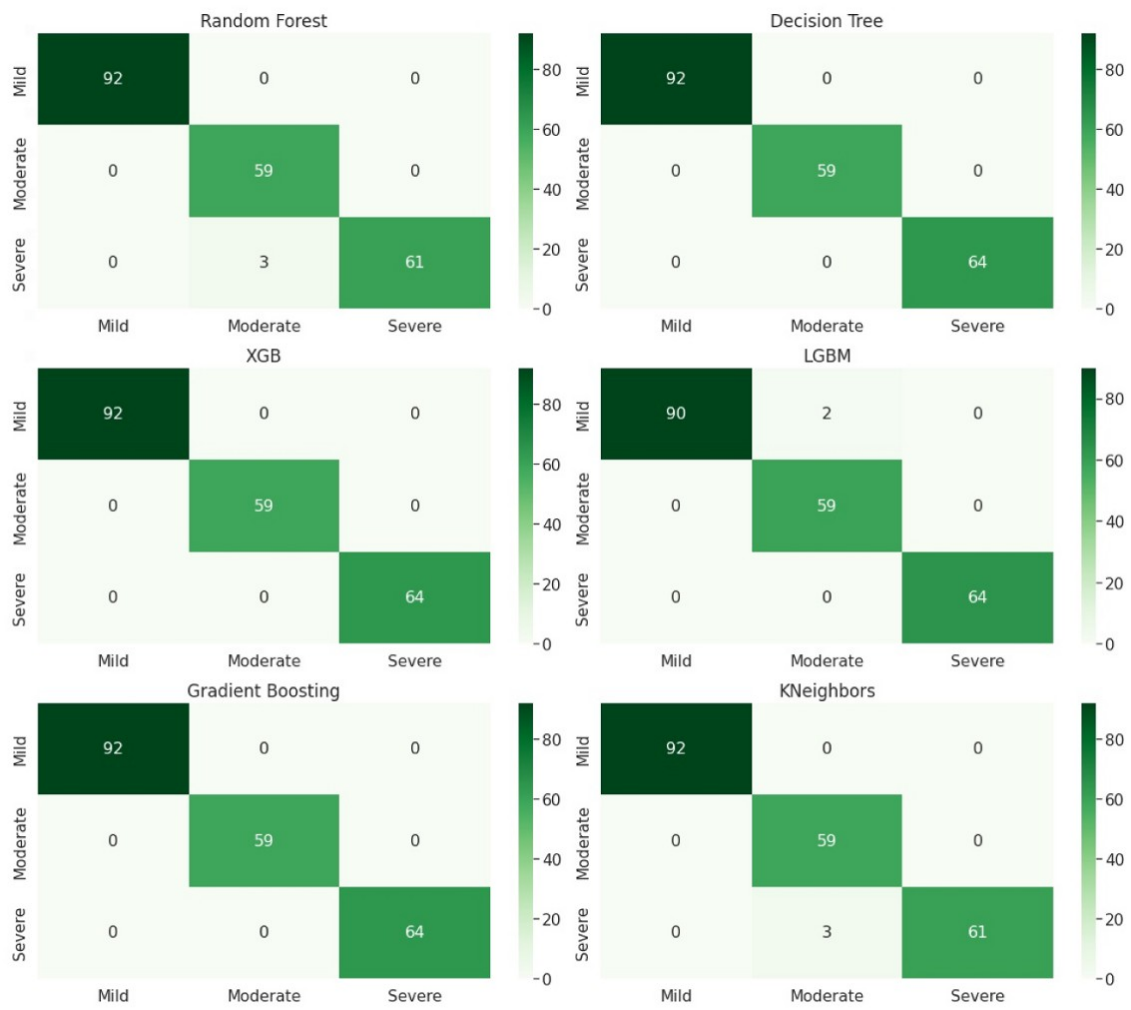
Classification of different classes of anxiety.

**Fig 4:**
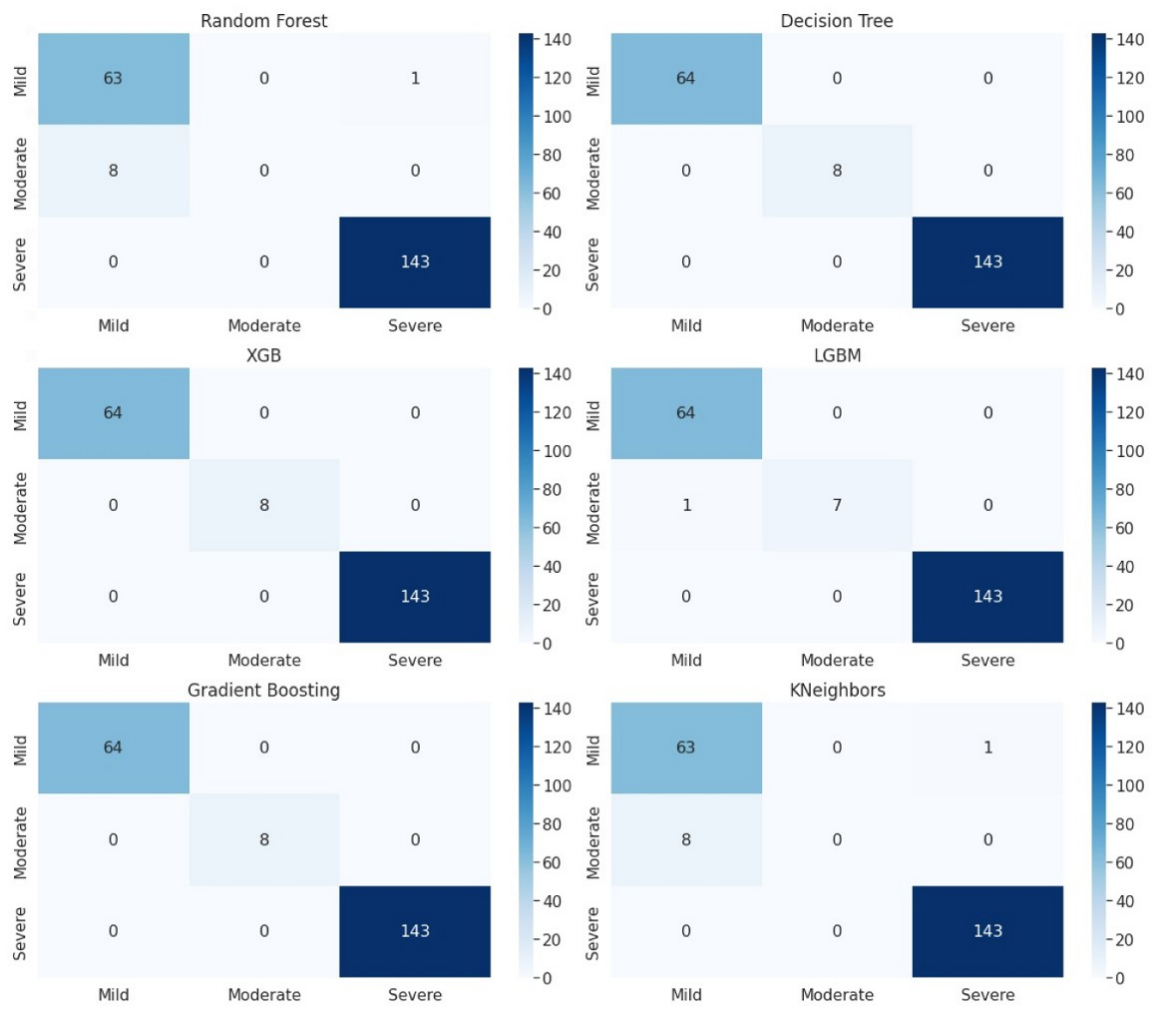
Classification of different classes of depression.

To better understand the performance of the classification algorithm, the confusion matrix was utilized in the study as shown in Figs 3 and 4. The accuracy values can be misleading if the dataset contains more than one class or unequal observations. The confusion matrix highlights the errors and provides a clear idea of the classification model’s results. The matrix summarizes the predicted results and indicates the accuracy percentage of the classification model. Although the matrix has several values, its main purpose is to identify where the machine learning models went wrong. The confusion matrix is created with two axes, with the test values of the dataset on the y-axis and the scale used to predict results for the dataset on the x-axis. The scale indicates three levels of intensity: mild, moderate, and severe. The machine learning algorithms predict different classes of the dataset, and the confusion matrix is drawn for both anxiety and depression classes. The confusion matrixes for anxiety and depression using algorithms such as Decision Tree, Random Forest, K Nearest Neighbor, Gradient Boosting, Extreme Gradient Boosting, and Light Gradient Boosting. The three-level scales depict the intensity of anxiety as severe, moderate, and mild.

### Anxiety classification reuslts

For classification of anxiety, Fig 3 is explaining the performance of proposed algorithms in the form of confusion matrix. We have three anxiety classes (mild, moderate, and severe). There are 92 instances of the mild class, 64 instances of the severe class, and 59 instances of the moderate class. The diagonal values represent the number of instances that are correctly classified for each class by the proposed algorithms. In order to further evaluate the performance of the model, additional metrics such as precision, recall, and F1-score are used to get a more comprehensive understanding of its performance as shown in Table 2.

Decision Tree, XGB, and Gradient Boosting algorithms are correctly classified 92 instances of the mild class, 59 instances of the moderate class, and 64 instances of the severe class. The number of wrong predictions of decesion tree, XGB and Gradient Boosting is zero, it means that all the instances in the dataset are classified correctly.

For KNN and random forest algorithms, there are 92 instances of the mild class, 59 instances of the moderate class, and 61 instances of the severe class that are correctly classified by these algorithms. It is important to note that the number of correctly classified instances for the moderate class is lower than that of the other two classes. There maybe an imbalance issue in moderate class. Similarly LightGBM shows that there are 90 instances of the mild class, 64 instances of the severe class, and 59 instances of the moderate class that are correctly classified. The results for LightGBM are also satisfactory.

### Depression classification reuslts

For classification of depression, Fig 4 is explaining the performance of proposed algorithms in the form of confusion matrix. We have three depression classes (mild, moderate, and severe). There are 64 instances of the mild class, 143 instances of the severe class, and 8 instances of the moderate class. The diagonal values represent the number of instances that are correctly classified for each class by the proposed algorithms. In order to further evaluate the performance of the model, additional metrics such as precision, recall, and F1-score are used to get a more comprehensive understanding of its performance as shown in Table 1.

The number of wrong predictions of decesion tree, XGB and Gradient Boosting is zero, it means that all the instances in the dataset are classified correctly.

Similarly, the confusion matrix for random forest and KNeighbors indicates that there are 63 instances of the mild class and 143 instances of the severe class that are correctly classified by the random forest and KNeighbors. However, the lower triangle value of 8 for the moderate class indicates that there are 8 instances that belong to the moderate class but were misclassified as belonging to another class by the random forest and KNeighbors. This means that random forest and KNeighbors are performing well but there is a class imbalance issue at moderate level.

Similarly LightGBM shows that there are 64 instances of the mild class, 143 instances of the severe class, and 7 instances of the moderate class that are correctly classified. The results for LightGBM are also satisfactory.

### Comparison with other approaches

For anxiety and depression detection, we have compared our finidings with other work as shown in Table 3. In [18], authors evaluated the performance of several machine learning algorithms on a publicly available dataset. They reported the accuracy of each algorithm, including DT (74.12%), RF (92.0%), k-NN(71.76%), and GB (82.35%). In [27], authors used a private dataset from the corresponding author and reported the accuracy of DT (86.7%) and RF (97.2%). In [28], they also used a private dataset and reported the accuracy of RF (73.7%), GB (64.6%), and XGB (66.3%). In [29], used a dataset called DASS-21 and reported the accuracy of DT (73%), RF (71%), and k-NN (69%). In [30], authors used an online dataset and reported the accuracy of DT (80.69%), (81.22%), and k-nearest neighbor (80.42%). Finally, the proposed study of depression detection on a public dataset and reported the accuracy of DT (100%), RF(96%), k-NN (96%), GB (100%), XGB (100%), and LGBM (99%). Similarly, the proposed study of anxiety detection on a public dataset and reported the accuracy of DT (100%), RF(97%), k-NN (97%), GB (100%), XGB (100%), and LGBM (99%).

**Table 3:** Comparison of our work with existing research in terms of accuracy.

References	Dataset	DT	RF	K-NN	GB	XGB	LGBM
[18]	Public	74.12%	92.0%	71.76%	82.35%	-	-
[27]	Private	86.7%	97.2%	-	-	-	-
[28]	Private	-	73.7%	-	64.6%	66.3%	-
[29]	Public	73%	71%	69%	-	-	-
[30]	Public	80.69%	81.22%	80.42%	-	-	-
Depression Detection	Public	100%	96%	96%	100%	100%	99%
Anxiety Detection	Public	100%	97%	97%	100%	100%	99%

The results of this study suggest that machine learning algorithms can be valuable in predicting mental health outcomes among healthcare workers during pandemics. The findings also highlight the need for mental health support for healthcare workers, especially in addressing the high levels of depression they face during pandemics.

## Conclusion

The COVID-19 pandemic has resulted in a significant impact on the mental health of healthcare workers globally. They are now more susceptible to depression and anxiety. In this study, we aimed to identify the relationship between the psychological impact of COVID-19 and several sociodemographic factors experienced by frontline health workers in Saudi Arabia. The dataset used in this study was collected through an online survey of medical health professionals. It consisted of 75 columns, out of which 58 were categorical columns. To analyze the dataset, we employed various machine learning algorithms, including decision tree, random forest, kNeighbors, gradient boosting, extreme gradient boosting, and light gradient boosting. Our analysis revealed that decision tree, gradient boosting, and extreme gradient boosting achieved 100% correct classification results for both anxiety and depression detection. However, random forest and kNeighbors misclassified instances with mild anxiety and moderate depression. Similarly, LGBM misclassified two instances of mild anxiety and one instance of moderate depression. Despite these misclassifications, all the algorithms’ accuracy rates are above 99% for anxiety and depression detection. Our results showed considerable rates of anxiety and depression among Saudi front-line workers. Our study’s intended purpose is to inform policy-makers about the importance of healthcare professionals’ mental health condition.

## References

[1] Wang C, Pan R, Wan X, Tan Y, Xu L, McIntyre RS, et al. A longitudinal study on the mental health of general population during the COVID-19 epidemic in China. Brain, behavior, and immunity. 2020 Jul 1;87:40–8. doi: 10.1016/j.bbi.2020.04.028 32298802 PMC7153528

[2] Xiang YT, Yang Y, Li W, Zhang L, Zhang Q, Cheung T, et al. Timely mental health care for the 2019 novel coronavirus outbreak is urgently needed. The lancet psychiatry. 2020 Mar 1;7(3):228–9. doi: 10.1016/S2215-0366(20)30046-8 32032543 PMC7128153

[3] “Coronavirus disease (COVID-19) Situation Report -116,” Who.int, 2020. [Online]. Available: https://www.who.int/docs/default-source/coronaviruse/situation-reports/20200515-covid-19-sitrep-116.pdf. [Accessed: 11-Oct-2022].

[4] Koh D, Lim MK, Chia SE, Ko SM, Qian F, Ng V, et al. Risk perception and impact of severe acute respiratory syndrome (SARS) on work and personal lives of healthcare workers in Singapore what can we learn?. Medical care. 2005 Jul 1:676–82. doi: 10.1097/01.mlr.0000167181.36730.cc 15970782

[5] Le XT, Nguyen QT, Onyango B, Nguyen QN, Pham QT, Ta NT, et al. Perception toward exposure risk of COVID-19 among health workers in Vietnam: Status and correlated factors. Frontiers in public health. 2021 May 25;9:589317. doi: 10.3389/fpubh.2021.589317 34113595 PMC8185209

[6] Dong Y, Yeo MC, Tham XC, Danuaji R, Nguyen TH, Sharma AK, et al. Investigating psychological differences between nurses and other health care workers from the Asia-Pacific region during the early phase of COVID-19: Machine learning approach. JMIR nursing. 2022 Jun 1;5(1):e32647. doi: 10.2196/32647 35648464 PMC9162133

[7] Chew NW, Ngiam JN, Tan BY, Tham SM, Tan CY, Jing M, et al. Asian-Pacific perspective on the psychological well-being of healthcare workers during the evolution of the COVID-19 pandemic. BJPsych open. 2020 Nov;6(6):e116. doi: 10.1192/bjo.2020.98 33028449 PMC7542327

[8] Chew NW, Lee GK, Tan BY, Jing M, Goh Y, Ngiam NJ, et al. A multinational, multicentre study on the psychological outcomes and associated physical symptoms amongst healthcare workers during COVID-19 outbreak. Brain, behavior, and immunity. 2020 Aug 1;88:559–65. doi: 10.1016/j.bbi.2020.04.049 32330593 PMC7172854

[9] Pham QT, Le XT, Phan TC, Nguyen QN, Ta NK, Nguyen AN, et al. Impacts of COVID-19 on the life and work of healthcare workers during the nationwide partial lockdown in Vietnam. Frontiers in Psychology. 2021 Aug 19;12:563193. doi: 10.3389/fpsyg.2021.563193 34489769 PMC8417359

[10] Rossi R, Socci V, Pacitti F, Di Lorenzo G, Di Marco A, Siracusano A, et al. Mental health outcomes among frontline and second-line health care workers during the coronavirus disease 2019 (COVID-19) pandemic in Italy. JAMA network open. 2020 May 1;3(5):e2010185-. doi: 10.1001/jamanetworkopen.2020.10185 32463467 PMC7256664

[11] Lee AM, Wong JG, McAlonan GM, Cheung V, Cheung C, Sham PC, et al. Stress and psychological distress among SARS survivors 1 year after the outbreak. The Canadian Journal of Psychiatry. 2007 Apr;52(4):233–40. doi: 10.1177/070674370705200405 17500304

[12] Lee SM, Kang WS, Cho AR, Kim T, Park JK. Psychological impact of the 2015 MERS outbreak on hospital workers and quarantined hemodialysis patients. Comprehensive psychiatry. 2018 Nov 1;87:123–7. doi: 10.1016/j.comppsych.2018.10.003 30343247 PMC7094631

[13] Fusaroli P, Balena S, Lisotti A. On the death of 100+ Italian doctors from COVID-19. Infection. 2020 Oct;48:803–4. doi: 10.1007/s15010-020-01436-1 32358774 PMC7193540

[14] Tan BY, Chew NW, Lee GK, Jing M, Goh Y, Yeo LL, et al. Psychological impact of the COVID-19 pandemic on health care workers in Singapore. Annals of internal medicine. 2020 Aug 18;173(4):317–20. doi: 10.7326/M20-1083 32251513 PMC7143149

[15] Wang C, Pan R, Wan X, Tan Y, Xu L, Ho CS, et al. Immediate psychological responses and associated factors during the initial stage of the 2019 coronavirus disease (COVID-19) epidemic among the general population in China. International journal of environmental research and public health. 2020 Mar;17(5):1729. doi: 10.3390/ijerph17051729 32155789 PMC7084952

[16] Albagmi FM, AlNujaidi HY, Al Shawan DS. Anxiety levels amid the COVID-19 lockdown in Saudi Arabia. International Journal of General Medicine. 2021 May 31:2161–70. doi: 10.2147/IJGM.S312465 34103971 PMC8180301

[17] Alyami HS, Naser AY, Dahmash EZ, Alyami MH, Alyami MS. Depression and anxiety during the COVID‐19 pandemic in Saudi Arabia: a cross‐sectional study. International journal of clinical practice. 2021 Jul;75(7):e14244. doi: 10.1111/ijcp.14244 33876864 PMC8249996

[18] Rezapour M, Hansen L. A machine learning analysis of COVID-19 mental health data. Scientific Reports. 2022 Sep 2;12(1):14965. doi: 10.1038/s41598-022-19314-1 36056129 PMC9438361

[19] Kotsiantis SB. Decision trees: a recent overview. Artificial Intelligence Review. 2013 Apr;39:261–83. doi: 10.1007/s10462-011-9272-4

[20] Breiman L. Classification and regression trees. Routledge; 2017 Oct 19.

[21] Criminisi A, Shotton J, Konukoglu E. Decision forests: A unified framework for classification, regression, density estimation, manifold learning and semi-supervised learning. Foundations and trends^®^ in computer graphics and vision. 2012 Mar 28;7(2–3):81–227.

[22] Jiang L, Cai Z, Wang D, Jiang S. Survey of improving k-nearest-neighbor for classification. InFourth international conference on fuzzy systems and knowledge discovery (FSKD 2007) 2007 Aug 24 (Vol. 1, pp. 679-683). IEEE.

[23] Qiao Z, Sun N, Li X, Xia E, Zhao S, Qin Y. Using machine learning approaches for emergency room visit prediction based on electronic health record data. InBuilding continents of knowledge in Oceans of data: The future of co-created eHealth 2018 (pp. 111-115). IOS Press. 29677933

[24] Ke G, Meng Q, Finley T, Wang T, Chen W, Ma W, et al. Lightgbm: A highly efficient gradient boosting decision tree. Advances in neural information processing systems. 2017;30.

[25] Chen T, Guestrin C. Xgboost: A scalable tree boosting system. InProceedings of the 22nd acm sigkdd international conference on knowledge discovery and data mining 2016 Aug 13 (pp. 785-794).

[26] Chen T, He T, Benesty M, Khotilovich V, Tang Y, Cho H, et al. Xgboost: extreme gradient boosting. R package version 0.4-2. 2015 Aug 1;1(4):1-4.

[27] Qasrawi R, Polo SP, Al-Halawa DA, Hallaq S, Abdeen Z. Assessment and prediction of depression and anxiety risk factors in schoolchildren: machine learning techniques performance analysis. JMIR Formative Research. 2022 Aug 31;6(8):e32736. doi: 10.2196/32736 35665695 PMC9475423

[28] Chung J, Teo J. Mental health prediction using machine learning: taxonomy, applications, and challenges. Applied Computational Intelligence and Soft Computing. 2022 Jan 5;2022:1–9. doi: 10.1155/2022/9970363

[29] Priya A, Garg S, Tigga NP. Predicting anxiety, depression and stress in modern life using machine learning algorithms. Procedia Computer Science. 2020 Jan 1;167:1258–67. doi: 10.1016/j.procs.2020.03.442

[30] Vaishnavi K, Kamath UN, Rao BA, Reddy NS. Predicting mental health illness using machine learning algorithms. InJournal of Physics: Conference Series 2022 (Vol. 2161, No. 1, p. 012021). IOP Publishing.

