# Correction: An Intelligent Framework to Measure the Effects of COVID-19 on the Mental Health of Medical Staff

**DOI:** 10.1371/journal.pone.0295459

**Published:** 2023-11-30

**Authors:** The PLOS ONE Staff

There are errors in the Funding statement and the publisher apologizes for the errors. The correct Funding statement is as follows: This work was funded by the Deanship of Scientific Research at Najran University, Kingdom of Saudi Arabia, under the Research Groups funding program with the grant code number NU/RG/SERC/12/9. The authors express their gratitude for this support.
